# The Spatiotemporal Stability of Dominant Frequency Sites in *In-Silico* Modeling of 3-Dimensional Left Atrial Mapping of Atrial Fibrillation

**DOI:** 10.1371/journal.pone.0160017

**Published:** 2016-07-26

**Authors:** Changyong Li, Byounghyun Lim, Minki Hwang, Jun-Seop Song, Young-Seon Lee, Boyoung Joung, Hui-Nam Pak

**Affiliations:** Yonsei University Health System, Seoul, Republic of Korea; Gent University, BELGIUM

## Abstract

**Background:**

We previously reported that stable rotors were observed in *in-silico* human atrial fibrillation (AF) models, and were well represented by dominant frequency (DF). We explored the spatiotemporal stability of DF sites in 3D-AF models imported from patient CT images of the left atrium (LA).

**Methods:**

We integrated 3-D CT images of the LA obtained from ten patients with persistent AF (male 80%, 61.8 ± 13.5 years old) into an *in-silico* AF model. After induction, we obtained 6 seconds of AF simulation data for DF analyses in 30 second intervals (T1–T9). The LA was divided into ten sections. Spatiotemporal changes and variations in the temporal consistency of DF were evaluated at each section of the LA. The high DF area was defined as the area with the highest 10% DF.

**Results:**

1. There was no spatial consistency in the high DF distribution at each LA section during T1–T9 except in one patient (p = 0.027). 2. Coefficients of variation for the high DF area were highly different among the ten LA sections (p < 0.001), and they were significantly higher in the four pulmonary vein (PV) areas, the LA appendage, and the peri-mitral area than in the other LA sections (p < 0.001). 3. When we conducted virtual ablation of 10%, 15%, and 20% of the highest DF areas (n = 270 cases), AF was changed to atrial tachycardia (AT) or terminated at a rate of 40%, 57%, and 76%, respectively.

**Conclusions:**

Spatiotemporal consistency of the DF area was observed in 10% of AF patients, and high DF areas were temporally variable. Virtual ablation of DF is moderately effective in AF termination and AF changing into AT.

## Introduction

Atrial fibrillation (AF) is the most common cardiac electrophysiological rhythm disturbance that results in the absence of normal atrial contractions. During the past decade, radiofrequency catheter ablation (RFCA) of AF has evolved rapidly from an investigational procedure to the standard procedure for antiarrhythmic drug resistant AF [1]. Current clinical ablation strategies are largely based on atrial anatomy and substrate detected using different approaches, and they differ from one clinical center to another [2]. Recently, Narayan et al. reported that detection and ablation of rotors in AF patients is effective in terminating AF and improves the clinical outcome of AF catheter ablation [3]. However, the detection of a mother rotor, which is stable and induces fibrillatory conduction [4–7], is affected by the spatiotemporal resolution of mapping and detection parameters. We recently simulated a mother rotor in 2-D and 3-D simulation models of human AF and documented the locations of the rotors, which were well represented by dominant frequency (DF) [8]. Nevertheless, it has been reported that the DF is temporally variable and that high DF sites can be transient in clinical experimentation [9–11]. Therefore, we explored the spatiotemporal stability of DF sites in patient-specific left atrium (LA) geometry-integrated *in-silico* modeling of human AF. Computer simulation modeling provides a unique advantage to evaluating the spatiotemporal variance from single cells to entire tissue regions under various conditions reproducibly and precisely [12,13]. The purpose of this study was to evaluate the spatiotemporal variability of high DF sites at nine specified periods in ten different LA sections among ten different patient-specific LA models of AF, as well as to assess the outcome of virtual ablation for high DF sites.

## Methods

The study protocol was approved by the Institutional Review Board of Severance Cardiovascular Hospital, Yonsei University Health System, and adhered to the Declaration of Helsinki. All subjects provided written informed consent.

### A. 3-D atrial remodeling

The 3-D *in-silico* model of the human LA was reconstructed using an by EnSite NavX^®^ system (Endocardial Solutions, St. Jude Medical, Inc., St. Paul, MN, USA) with computed tomographic (CT) image data from clinical persistent AF patients. Cellular ionic currents were calculated using the Courtemanche [14] human atrial myocyte model, and electrical wave conduction in tissue was simulated using the following partial differential Eq (1) [15]:

$$\frac{\partial V}{\partial t}=D\nabla^{2}V-\frac{I_{ion}+I_{stim}}{C_{m}},$$ (1)

where V is the membrane potential, D is the diffusion coefficient that represents gap junctional coupling, I_ion_ and I_stim_ are the total transmembrane ionic current and stimulus current, respectively, and C_m_ is the membrane capacitance of human atrial myocyte. AF modeling was implemented using CUDA 6.5 in Microsoft Visual Studio 10.0 (Microsoft Co., Redmond, WA, USA) for computer simulation. For the remodeling of ion currents of AF, we reduced I_to_, I_Kur_, I_CaL_ by 80%, 50%, and 40%, respectively [16,17], and increased I_K1_ by 50% [18]. Additionally, the diffusion coefficient was adjusted to simulate a conduction velocity (CV) of 0.4 m/s and an action potential duration at 90% repolarization (APD_90_) of 210–220 ms. We chose a conduction velocity of 0.4 m/s based on real human patient data (Yonsei AF ablation cohort data; n = 1,980; mean CV = 0.43 ± 0.24 m/s) [19]. For AF initiation, we used a series of localized stimulations that mimicked an experimental ramp pacing protocol [20]. Cells located near the LA high septum were stimulated at cycle lengths of 200, 190, and 180 ms consecutively (Straight Pacing Protocol). We applied the Runge-Kutta method with an adaptive time step of Δt = 0.005–0.05 ms and a generalized finite difference scheme on the LA surface mesh [21].

### B. DF generation and analysis algorithm

**Fig 1:**
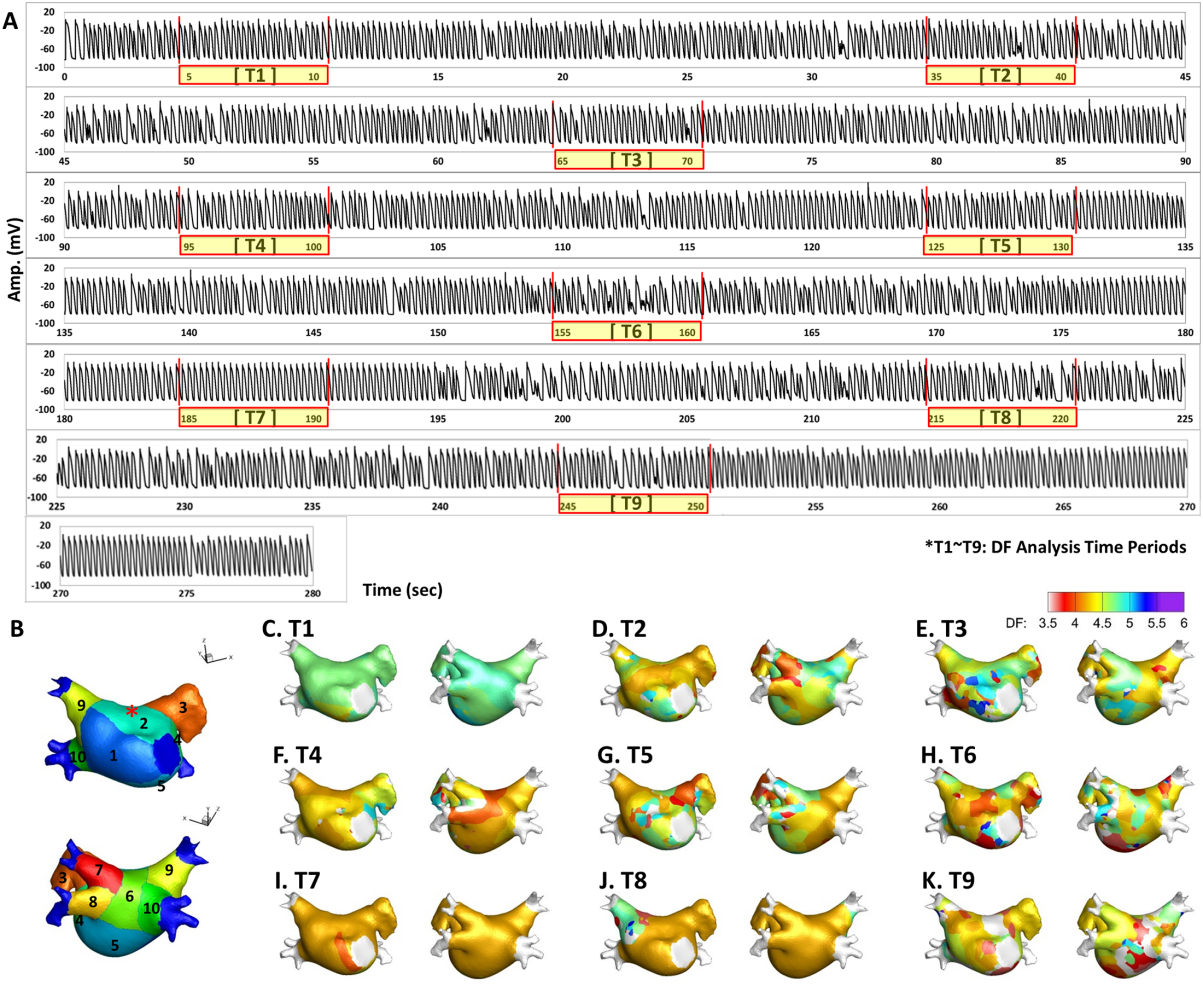
**A.** Electrogram (EGM) of action potential (AP) tracing for a total of 280 seconds and nine periods of DF analysis (T1–T9; 6 seconds in each period). **B.** Ten anatomical sections of the LA geometry. Asterisk: the node where AP tracing in panel A was acquired. **C~K.** Spatiotemporal changes of DF maps in a representative patient’s LA (T1–T9).

**Fig 2:**
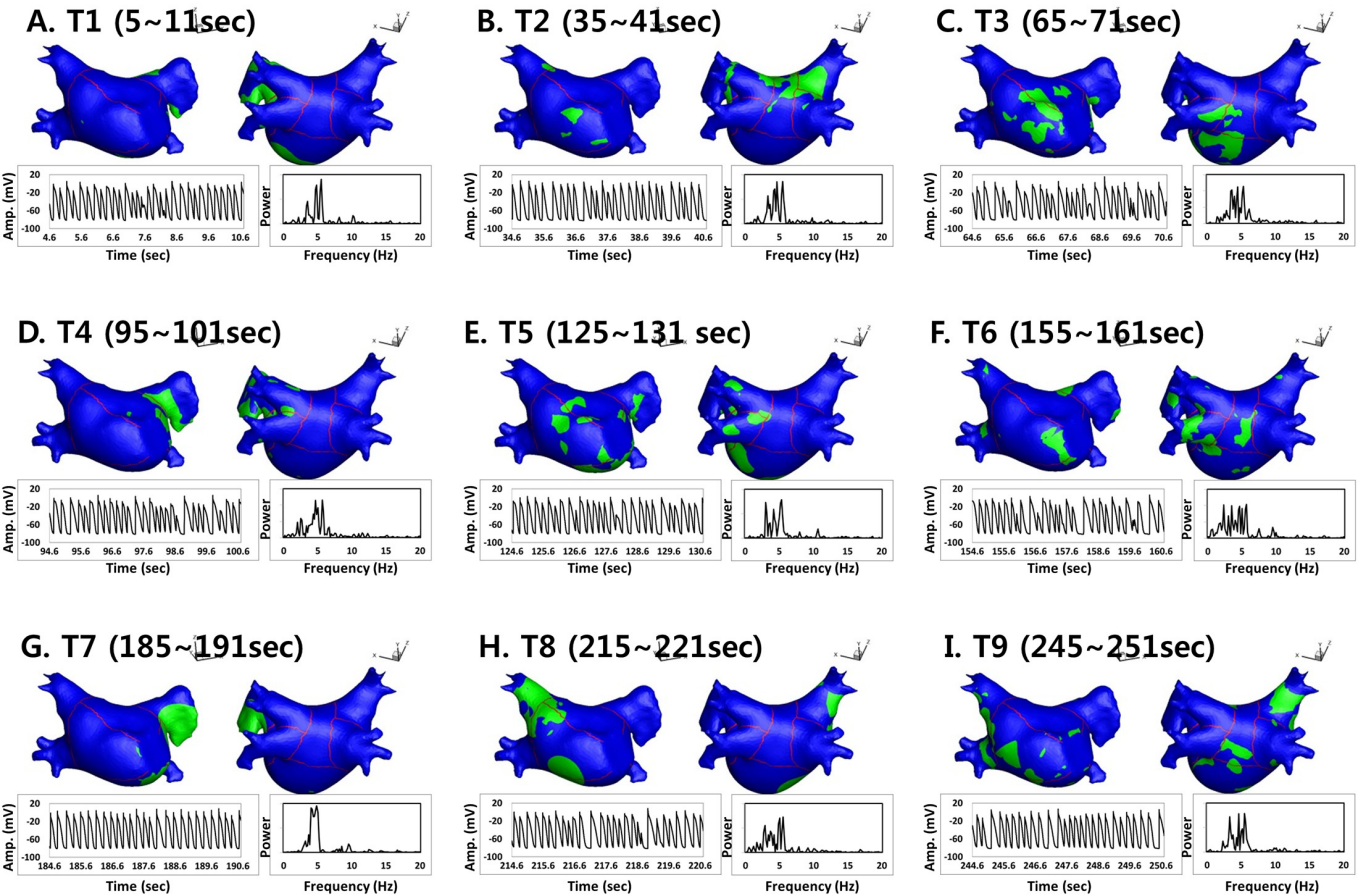
Spatiotemporal changes of the high DF area (≥ the highest 10% DF region) in each analysis period (T1–T9). AP tracing was acquired at the LA roof top, and the power spectrum of the fast Fourier transform analysis was obtained from the same AP tracing.

Using straight pacing (4560 ms), AF was induced and maintained, and we analyzed the spatiotemporal variability of the DF in AF lasting longer than 280 seconds. The electrogram (EGM) of the action potential (AP) for this process is shown in Fig 1A, and DF analysis time periods are expressed as T1 to T9. To determine the DF, the power spectral density was obtained via Fourier transform of the virtual action potential of each node, and the DF was defined as the frequency of the highest power [8]. We mapped the DF for 6 seconds at every 30 seconds during AF maintenance. To quantify the spatial distribution of the high DF area, we analyzed and compared ten different sections of the LA as shown in Fig 1B: R1, septum; R2, anterior wall; R3, LA appendage; R4, peri-mitral area; R5, posterior inferior wall; R6, posterior wall; R7–10, left upper and lower and right upper and lower pulmonary veins. An example of DF maps for the analysis time periods (T1–T9) is shown in Fig 1C–1K. We defined the “high DF area” as the region with the highest 10% of DF. As the area of each LA section was different, we calculated the regional proportion of the high DF area in each of the ten 10 LA sections. Fig 2 shows representative maps of a high DF (green) area (the highest 10% of the DF region), calculated with 6 seconds of AP for each node during periods T1 to T9.

### C. Virtual ablation for high DF area

**Fig 3:**
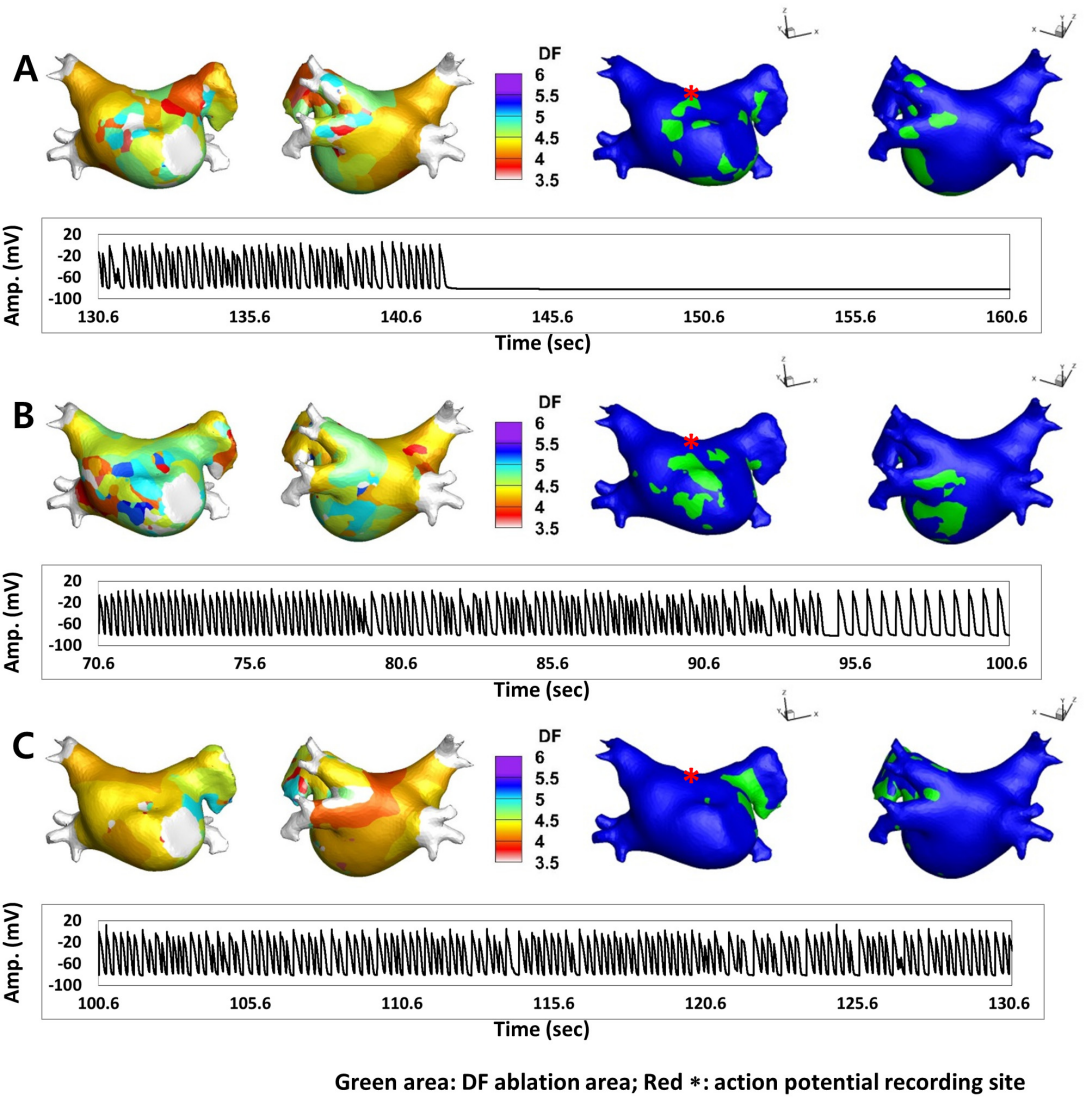
Examples of DF maps (left side maps), the highest 10% DF ablation maps (right side maps, green area) and AP tracings acquired from the LA roof top (red asterisk) after virtual DF ablation. **A.** AF was terminated at 142.2 seconds (11.6 seconds after virtual ablation of the high DF area). **B.** During DF ablation, AF changed into AT at 95.0 seconds (24.4 seconds after virtual ablation). **C.** The wave dynamics of AF did not change during DF ablation.

For virtual ablations, the conduction block was implemented by adjusting the diffusion coefficient parameter. The ablated region was set to the non-conduction condition to block the electrical conduction. An algorithm to detect the spatial distribution of the high DF area was implemented using MATLAB^®^ (MathWorks Inc., Natick, Massachusetts, USA). Virtual ablation was conducted at the end of each DF analysis period (T1–T9), and we observed the wave dynamics to determine whether AF terminated or changed to atrial tachycardia within 30 seconds after each ablation. The target of virtual ablation was the high DF area (10% highest DF value); however, we also performed virtual ablations for the 15% and 20% highest DF areas. Fig 3 shows examples of DF maps and the highest 10% DF ablation sites (green area). AP tracing acquired from the LA roof top (red asterisk) shows different responses after virtual DF ablation.

### D. Statistical analysis

Data are represented as mean ± standard deviation. The Friedman test was used to test the spatial variability of the DF values. A temporal coefficient of variation was used to assess the degree of temporal variation. Results of the temporal coefficient of variation analysis were analyzed using the Kruskal-Wallis test to detect the difference between each section. An independent-sample t-test was utilized to compare the mean temporal coefficient of variation among the four pulmonary vein (PV) areas, the LA appendage, and the peri-mitral area with that of the other sections. A chi-square test was used to analyze the differences in the variables between the trials with AF termination or AT conversion and those without AF change. For all analyses, p-values of < 0.05 were considered to be statistically significant. All data were analyzed with SPSS 19.0 statistical software (IBM Corporation, Somers, NY).

## Results

### A. Spatial consistency of the high DF area

**Fig 4:**
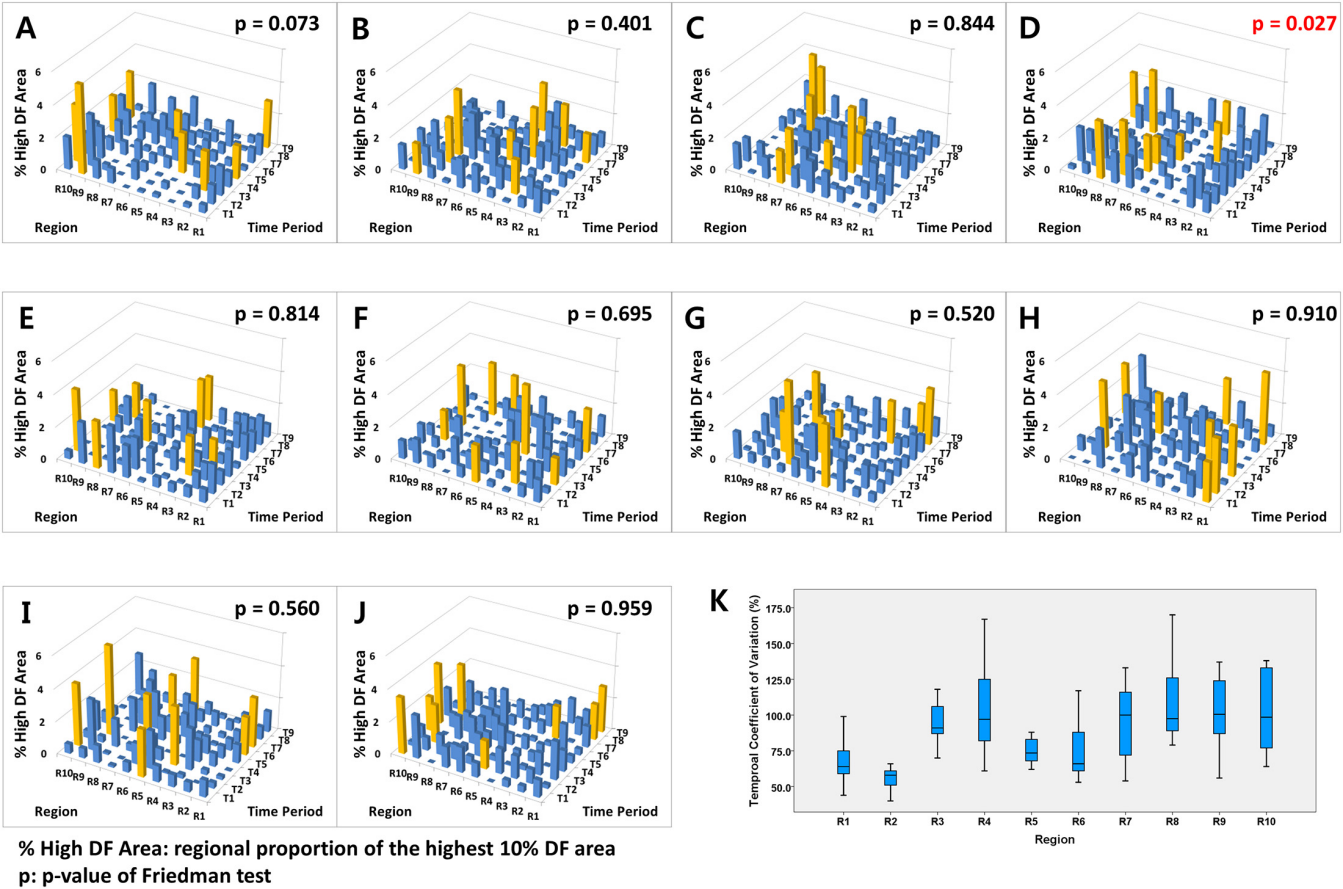
**A-J.** Variance of regional proportions of the high DF area (% High DF Area) in the ten LA sections (R1–R10) during the nine DF analysis periods (T1–T9) among ten patients. Yellow bars represent the highest % High DF Area among the ten LA sections. The % High DF Area represents the regional proportion of the highest 10% DF area (the highest 10% DF area / area of each LA section). R7–R10 represents the four pulmonary veins. The high DF area was consistently located in the pulmonary vein area in each patient (Panel 4D). **K.** The coefficients of variation from the overall analysis of the periods (T1–T9) in each region for all patients.

LA 3-D CT images obtained from 10 patients with persistent AF (age, 61.8 ± 13.5 years old; 80% male) were integrated into human AF modeling for DF analysis in this study. The characteristics of the patients are summarized in Table 1. The variances of the high DF area in the spatiotemporal distribution in each of the ten patients are shown for the ten different LA regions (R1–R10) during each of the nine different time periods (T1–T9; Fig 4). In Fig 4, the analysis time periods are marked from T1 to T9 on the x-axis, and the LA sections are represented from R1 to R10 on the y-axis. As the areas of each LA section are variable, we calculated the proportion of high DF area (% High DF Area; 10% (highest DF area / regional area) × 100), which is represented on the z-axis. Among the ten LA sections, the highest % High DF Area section is marked with yellow bars in Fig 4. Based on the Friedman test results, there was no spatial consistency in nine of the ten patients during the nine different time periods (p > 0.05, separately). However, one patient (10%, Fig 4D) showed a relatively consistent section of % High DF Area, mainly located in the pulmonary veins (R7–R10) during the time period (Friedman test, p = 0.027).

**Table 1:** Patients Characteristics.

**Age**	61.8 ± 13.5
**Male %**	80%
**Persistent AF, %**	100%
**CHA**_**2**_**DS**_**2**_**-VASc score**	11.5 ± 2.2
**Heart Failure, %**	0%
**Hypertension, %**	20%
**Age>75 years old, %**	20%
**Age 65–74 years old, %**	10%
**Diabetes, %**	30%
**Previous Stroke, %**	20%
**Previous TIA, %**	0%
**Vascular Disease, %**	30%
**LA dimension (mm)**	48.4 ± 7.9
**EF (%)**	59.2 ± 11.8
**E/Em**	11.5 ± 6.1

TIA, transient ischemic attack; LA, left atrium; EF, ejection fraction; E/Em, the ratio of early diastolic mitral inflow velocity (E) to early diastolic mitral annular velocity (Em).

### B. Spatiotemporal consistency of the high DF area in each LA section

The coefficient of variation (standard deviation / mean) was used to quantify the temporal variability for the regional proportion of the high DF area during the overall analysis time periods (T1–T9) in each LA section for all patients (Fig 4K). There was a significant difference in the coefficient of variation for each LA section during the overall DF analysis of the time periods. The average temporal coefficient of variation was 88.5 ± 27.7%. Comparison of the temporal coefficients of variation among the ten LA sections (R1–R10) also showed significant differences (p < 0.001). The temporal coefficients of variation for the % High DF Area were significantly higher in the four pulmonary vein areas (R7–R10), the LA appendage (R3), and the peri-mitral area (R4) than in the other LA regions (101.5 ± 25.9% vs. 69.0 ± 16.8%, p < 0.001).

### C. Virtual ablation for the high DF area

Although the high DF area seemed to be highly variable spatially and temporally in the majority of patients, we tested virtual ablation for the high DF area to examine whether the ablation terminated or defragmented AF (changing to atrial tachycardia) within 30 seconds after ablation. Virtual ablations were conducted on the 10%, 15%, and 20% highest DF sites in each time period (T1–T9) for each patient (overall 270 cases of high DF ablation). Fig 3 shows representative examples of the outcomes after virtual DF ablation. After virtual DF ablation, AF is terminated (Fig 3A), changed to organized atrial tachycardia (Fig 3B), or maintained (Fig 3C), depending on the AF conditions. Table 2 compares the outcome of virtual DF ablation depending on the extent of ablation. In the 10% highest DF ablation, AF was changed to AT or terminated in 40.0% of cases (36 of 90). In the 15% and 20% highest DF ablations, AFs were terminated or defragmented in 56.7% (51 of 90) and 75.6% (68 of 90), respectively (p < 0.001). It is unclear whether delayed AF termination or conversion to AT in 30 s was due to a DF site ablation effect or co-incidental wave-dynamic changes; nevertheless, the extent of DF ablation significantly affected the outcomes of virtual ablation.

**Table 2:** Outcome of Virtual Ablation for High DF Area depending on Extent of Ablation Area.

Definition	Percentage of DF ablation Area					
	10% ablation		15% ablation		20% ablation	
	N	(%)	N	(%)	N	(%)
AF maintenance	54	(60)	39	(43)	22	(24)
AF changed to AT	35	(39)	50	(56)	64	(71)
AF termination	1	(1)	1	(1)	4	(4)
	90	(100)	90	(100)	90	(100)

AT, atrial tachycardia

We varied the CV between 0.5 and 0.6 m/s and simulated AF induction and DF ablation. Although all induced AF lasted longer than 280 s at 0.4 m/s, AF was induced in 70% of cases via the same pacing protocol, although the AF terminated spontaneously within 33.5 ± 27.5 s at a CV of 0.5 m/s (p < 0.001 vs. 0.4 m/s). The AF induction rate was only 60% (six episodes) and induced AF self-terminated in 15.2 ± 7.3 s at a CV of 0.6 m/s (p < 0.001 vs. 0.4 m/s; S1 Fig). In these conditions, DF ablation could be attempted in only ten episodes at 0.5 m/s and in six episodes at 0.6 m/s. AF termination rates after DF ablation were significantly higher at CVs of 0.5 m/s and 0.6 m/s than at 0.4 m/s. However, all of the AF episodes at higher CV conditions were terminated itself without ablation (S1 Table). In the episodes in which DF ablation was followed by AF termination, the baseline AF maintenance duration (without ablation) was significantly shorter than in those without AF termination (73.7 ± 97.2 s vs. 231.5 ± 59.8 s, p < 0.001). Therefore, DF ablation was more likely to terminate AF under easily terminating AF conditions yet not under long-lasting sustained AF conditions.

## Discussion

In this study, we evaluated the spatiotemporal stability of DF during AF in an *in-silico* modeling of 3-D entire LA mapping. Spatiotemporal consistency of the high DF area was observed in only 10% of the AF models using the atrial geometries of patients. DF areas were temporally variable, particularly in the PV, LA appendage, and peri-mitral areas. Virtual ablation for the high DF area was moderately effective in the defragmentation of AF.

### A. Rotor represented by DF in the *in-silico* model

Stable rotors have been considered as a mechanism of AF initiation and maintenance anatomically or functionally for the past few decades [5,6,22–28]. Large amounts of experimental evidence and *in-silico* validations have supported the role of stable rotors in AF [6,26,27]. The rotor area in the LA exhibits a dominant peak in the frequency spectra in experimental models of AF [22], and the high DF area is used to localize the source of AF in clinical settings [23]. In our recent *in-silico* study, the area of the highest DF coincided with the stable rotor center, and virtual ablation targeting the stable rotor was effective in terminating AF or changing AF to atrial tachycardia [29]. However, we found that the spatiotemporal stability of the high DF area was maintained in only a limited number of patients in the current study. As rotors often meander on the atrial wall, high DF sites corresponding to meandering rotors would meander as well. Despite this spatiotemporal variability of the high DF area, high DF area ablation was nevertheless effective in the termination or defragmentation of AF.

### B. Spatiotemporal variability of DF

Many experimental studies have shown that rotors are a very likely source of AF, and the central areas of rotors exhibit high DF [30]. In the current study, DF ablation in each time period induced significant changes in AF maintenance. “Moreover, the temporal variation of the high DF area was more significant in the pulmonary veins, LA appendage, and peri-mitral areas, which are known to frequently harbor AF sources [31–33]. Therefore, the main hurdle for rotor or DF ablation might be the mapping technique for migratory sources of AF. AF wave dynamics and the spatiotemporal consistency of DF are very much dependent on the characteristics of ion currents [13] or the degree of substrate remodeling.

Spatiotemporal instability of the DF site was relatively high in our models, and few episodes of AF were terminated by virtual DF site ablation (1–4%). Although the spatiotemporal instability of the DF site can be affected by the degree of electrical remodeling (ion current states) or critical mass (atrial size, presence of linear ablation, or PV isolation), the extreme spatiotemporal instability of AF may preclude the development of AF ablation strategies based on focal ablation in the atrium.

Although there have been clinical reports of successful rotor-guided ablation in humans AF [34], there is a degree of controversy regarding the limitations of the spatiotemporal resolution of rotor mapping [35] and reproducibility [36]. In the TOPERA mapping studies [37], the majority of patients had a history of AF ablation and the electroanatomical substrates were different from those of *de novo* ablation [37–38]. The recent RADAR-AF trial failed to prove the superiority of DF-guided ablation outcomes compared to those of conventional ablation [39], and AF termination rates with DF-guided ablation were very low in clinical conditions [40]. However, sequential electrogram acquisition may raise concerns about DF stability, and high DF sites have been reported to be spatiotemporally unstable in clinical settings [10–11,41]. An alternative is that DF assessment in clinical conditions can differ greatly due to bipolar electrogram characteristics; therefore, DF computation using standard methods may not always accurately summarize the local rate of activation [42].

We observed reductions of AF induction rate and AF maintenance duration with slightly increased CV (0.5 and 0.6 m/s). It is because the wavelength of activation fronts was not sufficient to sustain the reentry and the wavefronts eventually die out by running into the area of refractoriness. Therefore, AF seems to be terminated mostly due to itself and not due to ablation. The ablation potentially changes the wavefront dynamics rather than directly affecting AF termination or conversion to AT.

### C. Clinical implications of virtual DF ablation

Computer simulations have been increasingly utilized in the many fields of clinical medicine. The strengths of computer simulation modeling are the predictability of outcome after certain interventions and the ability to define the best strategy after reversible trials and errors [43–45]. Therefore, personalized AF simulation modeling might be effective in determining the proper target for AF ablation as a part of precision medicine. Ganesan et al. [46] demonstrated stable AF rotors using Shannon entropy mapping in bipolar electrograms, and Haissaguerre et al. [47] mapped the AF driver domain via non-invasive panoramic mapping with a body-surface electrode array. However, given that the spatial resolutions and rotor detection algorithms differ among clinical studies, the definitions of rotors and outcomes of AF rotor ablation have not been consistent despite its evident role in the maintenance mechanism of AF. Therefore, *in-silico* detection of DF and virtual ablation can be valuable in determining the target for AF termination [48] or predicting the risk of arrhythmia [49] in a personalized heart model. Although our current *in-silico* AF model is an oversimplified homogeneous simulation integrating patient-specific anatomy, we will likely be able to reflect atrial histology [50] and deduct patient-specific wave-dynamics via machine learning techniques [51] in the future, creating a simultaneous entire atrial mapping system.

### D. Limitations

Although we adopted patient-specific LA geometry, our 3-D *in-silico* model was a structurally homogeneous LA model. Hansen et al. [52] recently suggested a 3D full-thickness atrial model including the endocardium and epicardium; however, the current study was conducted using a simple surface mesh model. Thus, bi-atrial application, thickness variation, fiber orientation, and regional pathology or local electrophysiology could all affect wave propagation. However, wave propagation in the monolayer model was reported to be similar to that in a bilayer model except for the area of abrupt change of fiber orientation [53]. Additionally, the ionic current properties were spatially uniform in the current model, and spatial heterogeneity in the ionic current properties would have affected the wave dynamics. As the sections of the LA were divided manually based on a clinical ablation strategy, the size and shape of each LA section was not uniform.

## Conclusion

Although DF may localize rotors, its spatiotemporal consistency was observed in only 10% of AF cases. The Most temporally variable high DF areas were located on the pulmonary vein, LA appendage, or peri-mitral areas. Although the high DF area changed spatiotemporally, virtual ablation for high DF areas remains effective in the defragmentation of AF, including AF termination or changing into AT.

## Supporting Information

S1 Fig Self-limited AF episodes at higher CVs (upper panel) and outcomes of 10% DF ablations (lower panel). (TIF)

S1 Table Outcomes of virtual ablation for high DF area for CV 0.5 m/s and CV 0.6 m/s. (DOCX)
