# Supplementary figures and images for: The Spatiotemporal Stability of Dominant Frequency Sites in In-Silico Modeling of 3-Dimensional Left Atrial Mapping of Atrial Fibrillation
Source: PLoS One. 2016 Jul 26;11(7):e0160017. doi: 10.1371/journal.pone.0160017 (PMC4961424; doi:10.1371/journal.pone.0160017)

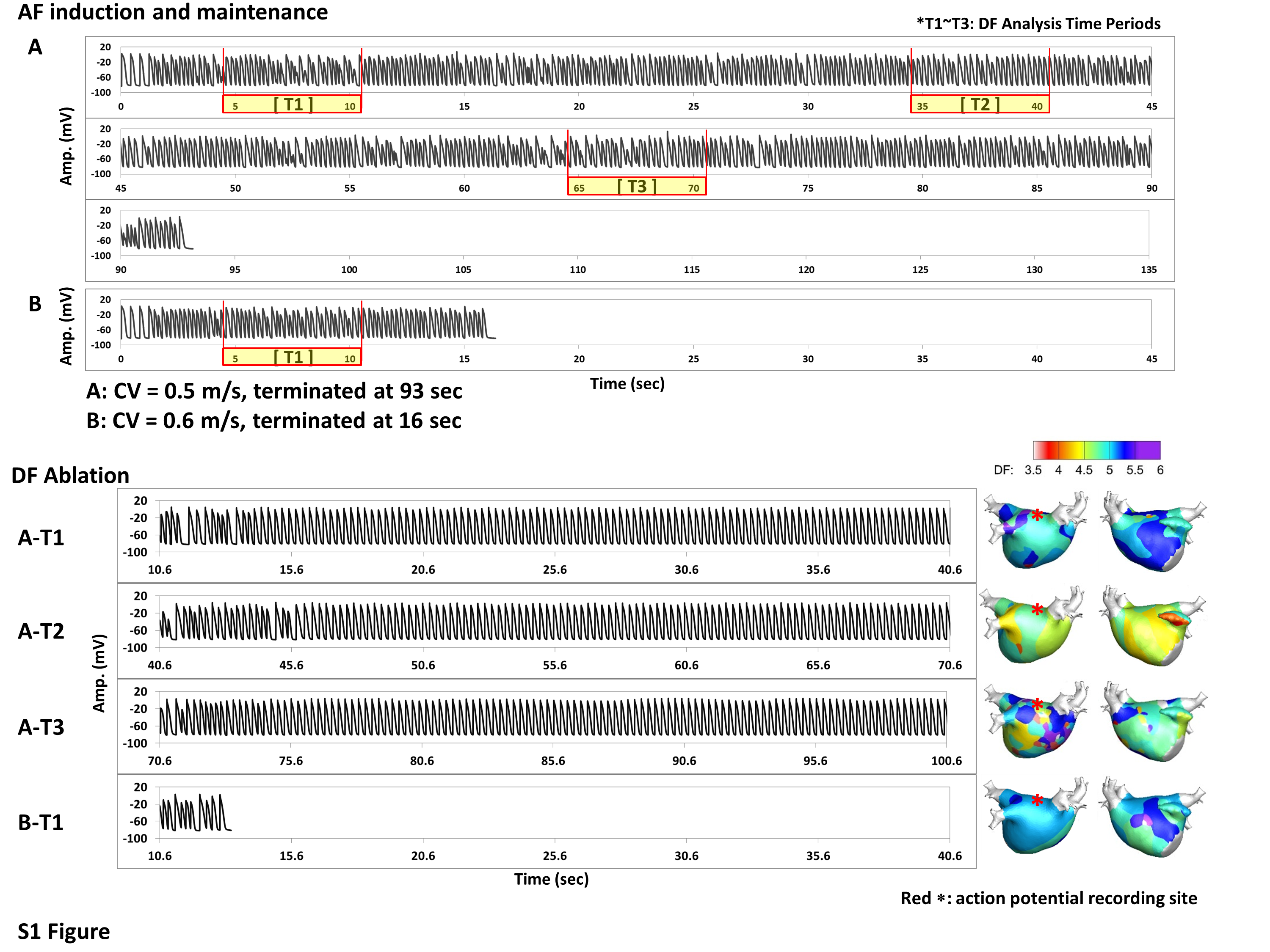

Supplement: S1 Fig — (TIF) [file pone.0160017.s001.tif]
