# Supplementary material for: The Spatiotemporal Stability of Dominant Frequency Sites in In-Silico Modeling of 3-Dimensional Left Atrial Mapping of Atrial Fibrillation
Source: PLoS One. 2016 Jul 26;11(7):e0160017. doi: 10.1371/journal.pone.0160017 (PMC4961424; doi:10.1371/journal.pone.0160017)
Supplement: S1 Table — (DOCX) [file pone.0160017.s002.docx]

**S1 Table.** Outcomes of virtual ablation for high DF area for CV 0.5 m/s and CV 0.6 m/s.

| **CV = 0.5 m/s** | **Percentage of DF ablation Area** | | | | | |
| --- | --- | --- | --- | --- | --- | --- |
|  | **10% ablation** | | **15% ablation** | | **20% ablation** | |
|  | **N** | **(%)** | **N** | **(%)** | **N** | **(%)** |
| AF maintenance | 0 | (0) | 0 | (0) | 0 | (0) |
| AF changed to AT | 8 | (80) | 4 | (40) | 6 | (60) |
| AF termination | 2 | (20)‡ | 6 | (60)* | 4 | (40) † |
|  | 10 | (100) | 10 | (100) | 10 | (100) |
|  | | | | | | |
| **CV = 0.6 m/s** | **Percentage of DF ablation Area** | | | | | |
|  | **10% ablation** | | **15% ablation** | | **20% ablation** | |
|  | **N** | **(%)** | **N** | **(%)** | **N** | **(%)** |
| AF maintenance | 0 | (0) | 0 | (0) | 0 | (0) |
| AF changed to AT | 2 | (33) | 3 | (50) | 3 | (50) |
| AF termination | 4 | (67)* | 3 | (50)* | 3 | (50) † |
|  | 6 | (100) | 6 | (100) | 6 | (100) |

AT: atrial tachycardia, *, p < 0.001 vs. CV 0.4 m/s counterpart; †, p < 0.01 vs. CV 0.4 m/s counterpart; ‡, p < 0.05 vs. CV 0.4 m/s counterpart in Table 2.
